# Particulate matter 2.5 causally increased genetic risk of autism spectrum disorder

**DOI:** 10.1186/s12888-024-05564-y

**Published:** 2024-02-16

**Authors:** Tianyu Jin, Qiongyi Pang, Wei Huang, Dalin Xing, Zitian He, Zheng Cao, Tong Zhang

**Affiliations:** 1 Department of Rehabilitation Medicine, The Second Affiliated Hospital and Yuying Children’s Hospital of Wenzhou Medical University, Wenzhou, Zhejiang China; 2 Department of Neurological rehabilitation, Beijing Bo’ai Hospital, China Rehabilitation Research Center, Beijing, China; 3 The Affiliated Drum Tower Hospital of Nanjing University Medical School, Nanjing, China; 4 Drum Tower Clinical Medical College, Nanjing Medical University, Nanjing, China; 5 Department of Medicine and Health, University of Sydney, Sydney, Australia

**Keywords:** Air pollution, Particulate matter, Autism spectrum disorder, Mendelian randomization, Risk

## Abstract

**Background:**

Growing evidence suggested that particulate matter (PM) exhibit an increased risk of autism spectrum disorder (ASD). However, the causal association between PM and ASD risk remains unclear.

**Methods:**

We performed two-sample Mendelian randomization (MR) analyses, using instrumental variables (IVs) sourced from the largest genome-wide association studies (GWAS) databases. We employed three MR methods: inverse-variance weighted (IVW), weighted median (WM), and MR-Egger, with IVW method serving as our primary MR method. Sensitivity analyses were performed to ensure the stability of these findings.

**Results:**

The MR results suggested that PM_2.5_ increased the genetic risk of ASD (β = 2.41, OR = 11.13, 95% CI: 2.54–48.76, *P* < 0.01), and similar result was found for PM_2.5_ absorbance (β = 1.54, OR = 4.67, 95% CI: 1.21–18.01, *P* = 0.03). However, no such association was found in PM_10_ (β = 0.27, OR = 1.30, 95% CI: 0.72–2.36, *P* = 0.38). After adjusting for the false discovery rate (FDR) correction, our MR results remain consistent. Sensitivity analyses did not find significant heterogeneity or horizontal pleiotropy.

**Conclusions:**

Our findings indicate that PM_2.5_ is a potential risk factor for ASD. Effective strategies to mitigate air pollutants might lead to a reduced incidence of ASD.

**Supplementary Information:**

The online version contains supplementary material available at 10.1186/s12888-024-05564-y.

## Introduction

Autism spectrum disorder (ASD) is a neurodevelopmental disorder characterized by impairments in social interaction, communication, and the presence of restricted interests and repetitive behaviors [1]. Recent epidemiological study has shown that 1 in 54 children in the United States is diagnosed with ASD, with a global prevalence of approximately 1% [2]. The disease presents significant socio-economic burdens, often stemming from lifelong rehabilitation and care needs, lost productivity, and the challenges related to the integration of affected individuals into society [2, 3]. The risk factors associated with ASD are not fully understood and appear to be related to genetic and environmental factors, such as family history, older parental age, pregnancy complications, and air pollution [4].

Air pollution presents a significant economic and social challenge worldwide, affecting both developed and developing nations. It is linked to a wide array of adverse health outcomes [5, 6]. Numerous studies have shown that air pollution is associated with a variety of diseases, such as cancer, respiratory diseases, cardiovascular diseases, and neurological diseases [7–10]. This extensive impact underscores the urgent need for a comprehensive understanding of air pollution’s global health implications, including its role in the etiology of ASD. Recent comprehensive meta-analysis illuminated a concerning correlation: particulate matter 2.5 (PM_2.5_) concentrations appear to increase the risk of ASD [11]. However, some studies did not find a significant relationship between PM_2.5_ and ASD [12, 13]. Additionally, the evidence regarding the effects of PM_10_ on ASD remains inconclusive [14]. Therefore, no study to date has conclusively determined the causal relationship between PM and ASD risk.

Traditional observational studies, including cohort, case-control, and cross-sectional studies, are crucial in epidemiology and medical research for understanding health outcomes, disease prevalence, and associations between risk factors and diseases. However, they have several limitations that can affect the validity and interpretation of their findings, such as residual confounding, reverse causation, and measurement error [15]. With the recent increase in availability of genome-wide association studies (GWAS) databases, Mendelian randomization (MR) is a novel method that utilizes genetic variants as instrumental variables (IVs) to infer causal relationships between potentially risk factors and diseases, based on the principles of Mendelian inheritance [16]. The strength of Mendelian randomization lies in its resemblance to randomized controlled trial (RCT). In RCT, participants are randomly assigned to receive a treatment or a placebo to establish causal relationships. Similarly, in MR analysis, the random allocation of genetic variants at conception is utilized to mimic the randomization process, helping to reduce confounding and reverse causation issues that often affect traditional observational studies [17, 18]. Consequently, MR provides a more robust approach to causal inference in epidemiological studies, potentially guiding public health interventions and therapeutic strategies. Additionally, it enables exploration of potential causality when RCTs are neither feasible nor ethical [17]. For instance, assigning individuals to harmful pollution exposure is unethical in a trial. Furthermore, this method has been widely employed to investigate causal relationships between PM_2.5_ and various diseases, including cancer [19], cardiovascular disease [20], thyroid diseases [21], and gestational diabetes [22].

In this study, we employed a two-sample MR analyses to investigate the potential association between PM and the risk of ASD.

## Methods

### Ethical approval

This MR study used published and publicly available GWAS data. Each participant received ethical approval and informed consent for the respective study, as detailed in the original publication and consortium.

### Study design

**Fig. 1:**
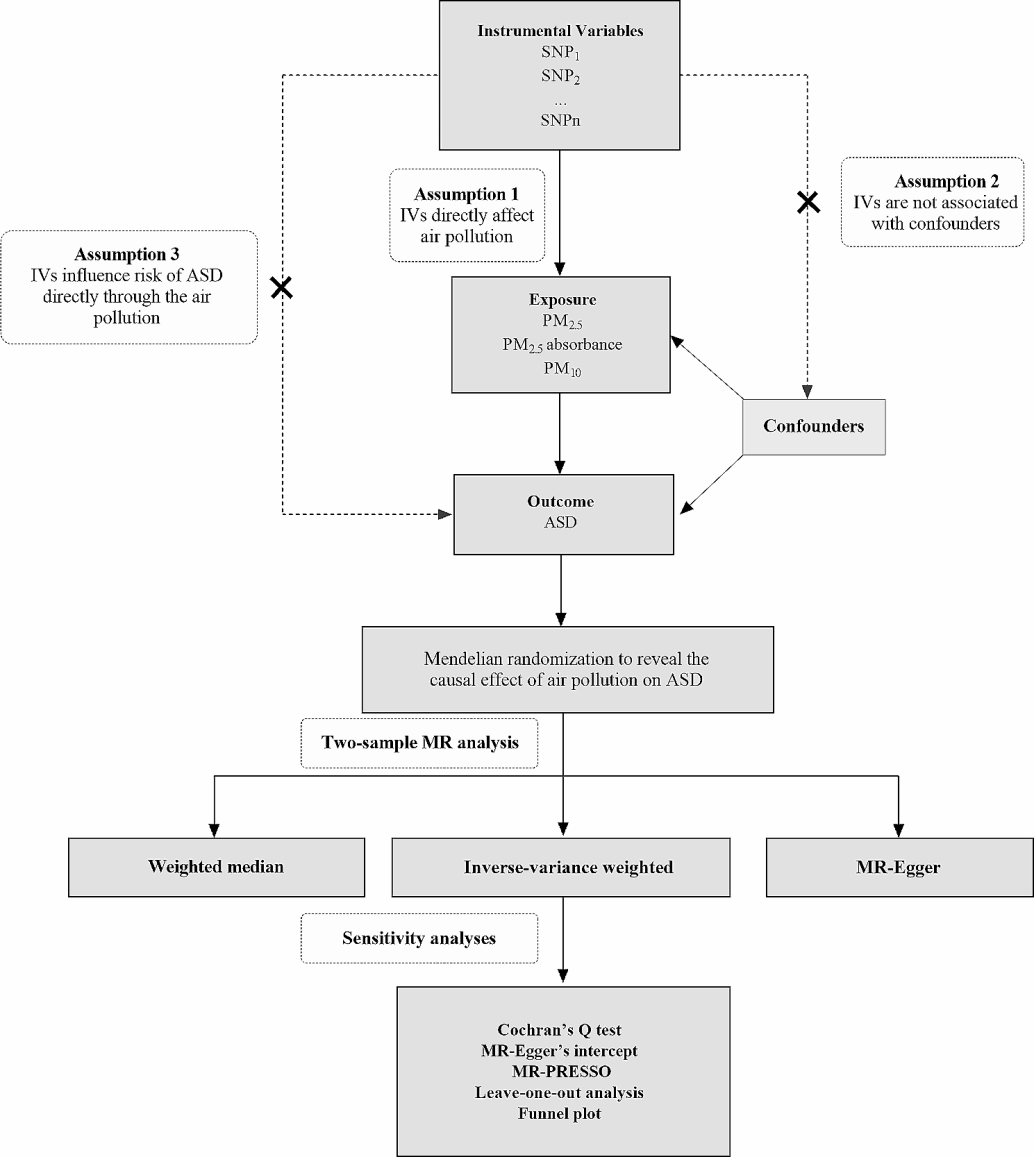
An overview of the study design

Our MR study followed the STROBE-MR statement [23]. In this study, we employed two-sample MR analysis. The exposure of interest was air pollution (PM_2.5_, PM_2.5_ absorbance and PM_10_), and the outcome was ASD. The three foundational hypotheses of MR are as follows [24]: (1) The genetic variant should be robustly associated with the air pollution. (2) The genetic variant should not be associated with any confounders of the air pollution-ASD relationship. (3) The genetic variant should influence the ASD only through its effect on the air pollution, and not via other pathways. Figure 1 illustrates our study design and the three core principles of MR analysis.

### Data source

The sources of datasets included in this MR study are listed in Table 1. For the exposure variable, we sourced our data from the UK Biobank [25]. The annual association between PM and residential addresses was analyzed using a Land Use Regression model from the European Study of Cohorts for Air Pollution Effects (ESCAPE). All the included populations were of European descent. The sample sizes were 423,796 individuals for both PM_2.5_ and PM_2.5_ absorbance, and 455,314 individuals for PM_10_. For ASD, we sourced the genetic association summary statistics from the iPSYCH-PGC consortium [26]. This dataset comprised 46,351 individuals of European descent, with 18,382 cases and 27,969 controls. Genotyping of the participants was conducted using case-cohort samples, identifying a cumulative 9,112,386 SNPs. The diagnosis of ASD was established using ICD-10 criteria.

**Table 1:** Details of the GWAS included in the mendelian randomization

Trait	Population	Sample size	Consortium/DOI
ASD	European	46,351	iPSYCH-PGC
PM_2.5_	European	423,796	UK biobank
PM_2.5_ absorbance	European	423,796	UK biobank
PM_10_	European	455,314	UK biobank

PM, particulate matter; ASD, autism spectrum disorder; GWAS, Genome-Wide Association Studies

### Selection of genetic instrumental variables

In this study, the selection of genetic IVs was guided by rigorous criteria to ensure the robustness and validity of our MR analyses. We selected genetic variants that reached a genome-wide significance level (*P* < 5 × 10^− 8^) [27]. To address concerns of collinearity and to guarantee the independence of instruments, we enforced a strict linkage disequilibrium (LD) criterion (*r*^2^ < 0.001) [28]. Moreover, we calculated the F-statistics to further ascertain the strength and validity of the instruments, which minimized the risk of weak instrument bias [29]. Furthermore, we considered proxy SNPs with a Proxy R^2^ > 0.8 when direct SNPs were unavailable, ensuring the proxies closely represented the genetic variation of interest. Additionally, we used PhenoScannerV2 to address potential confounding effects on the outcome at https://www.phenoscanner.medschl.cam.ac.uk.

### Mendelian randomization analyses

Three MR methods were used in our study, including inverse-variance weighted (IVW) method, weighted median (WM) method, and MR-Egger method [15, 24]. The IVW method is the primary method for MR. It provides a weighted average of the individual causal effect estimates derived from each genetic variant, with weights inversely proportional to the variance of the genetic associations with the outcome. This method assumes that all instruments are valid, implying no horizontal pleiotropy [15]. Essentially, the IVW method is a weighted linear regression model, treating each effect estimate of the genetic variant as a separate observation, with the weights being the inverse of the variance of these estimates [30, 31]. The WM method provides a consistent estimate of the causal effect even if up to 50% of the weight in the analysis comes from invalid instruments. By ordering the ratio estimates and their corresponding weights, this method determines a median value, offering a robustness to certain levels of invalid IVs [32]. The MR-Egger method permits the intercept to be non-zero, which captures the average pleiotropic effect across genetic variants. A non-zero intercept indicates potential directional pleiotropy. The slope of MR-Egger provides a causal effect estimate that is corrected for pleiotropy under the assumption of the Instrument Strength Independent of Direct Effect (InSIDE) condition [33]. In addition, we calculated the false discovery rate (FDR) adjusted *P* values to account for multiple testing, deeming those with a q-value of less than 0.05 to be statistically significant [34].

### Sensitivity analyses

Cochran’s Q test is applied to assess heterogeneity among the individual causal estimates derived from different genetic variants. A significant heterogeneity can indicate potential invalid instruments [35]. MR-Egger’s intercept provides a test for the presence of horizontal pleiotropy. A significant intercept suggests that some genetic variants might be invalid instruments due to their influence on the outcome independent of the exposure, which can bias the MR estimate [15]. Mendelian Randomization Pleiotropy Residual Sum and Outlier (MR-PRESSO) is designed to identify and correct for pleiotropic outliers. It enhances the reliability of the causal estimate by removing genetic variants that display horizontal pleiotropy [36]. Leave-one-out analysis involves systematically removing one genetic variant at a time from the analysis and recalculating the causal estimate. This process assesses whether the results are overly reliant on a particular variant or a set of variants and ensures that no single variant unduly influences the overall causal estimate [37]. Funnel plots visually assess the symmetry of the individual variant causal effect estimates around the combined MR estimate. Asymmetry in the funnel plot could be indicative of pleiotropy or other violations of the standard MR assumptions [38]. Finally, we compute the power of our MR analyses using the platform available at https://shiny.cnsgenomics.com/mRnd.

## Results

### Genetic instruments variables for air pollution

For IVs of air pollution, we included 8 SNPs associated with PM_2.5_, 5 SNPs associated with PM_2.5_ absorbance, and 22 SNPs related to PM_10_, each of these had an F-statistic greater than 10. Moreover, no SNPs were found to be associated with risk factors for ASD using PhenoScanner V2. During the harmonization process, the SNPs rs114708313, rs140295641, rs6867849, and rs74805019 were removed because of their palindromic nature with intermediate allele frequencies or due to incompatible alleles. Detailed characteristics of the IVs for particulate matter air pollution can be found in Table S1.

### Mendelian randomization analysis for causal association of PM_2.5_ and ASD

Detailed MR estimates and sensitivity analyses are provided in Table 2.

**Table 2:** The Mendelian randomization of particulate matter air pollution on autism spectrum disorder risk

Exposures	Mendelian randomization estimates					Sensitivity analyses	
	Method	β	OR	95% CI	*P* value	Heterogeneity (Q_P value)	MR-Egger’s intercept (*P* value)
	MR-Egger	5.67	290.28	0.12-6.61e^5^	0.22	< 0.01*	-0.0623 (0.34)
PM_2.5_	Weighted median	2.03	7.62	1.74–33.29	0.01*		
	Inverse variance weighted	1.51	4.54	0.55–37.67	0.16	< 0.01*	
	MR-Egger	4.30	73.51	0.30-1.78e^4^	0.22	0.07	-0.0295 (0.53)
PM_2.5_ (adjusted)	Weighted median	2.60	13.47	3.14–57.82	< 0.01*		
	Inverse variance weighted	2.41	11.13	2.54–48.76	< 0.01*	0.08	
	MR-Egger	0.95	2.60	0.06-110.75	0.65	0.12	0.0123 (0.76)
PM_2.5_ absorbance	Weighted median	1.38	3.97	0.86–18.29	0.08		
	Inverse variance weighted	1.54	4.67	1.21–18.01	0.03*	0.20	
	MR-Egger	-0.74	0.48	0.06–3.74	0.49	0.05*	0.0215 (0.24)
PM_10_	Weighted median	0.17	1.18	0.52–2.66	0.69		
	Inverse variance weighted	0.47	1.60	0.79–3.24	0.19	0.04*	
	MR-Egger	-0.16	0.85	0.14–5.14	0.86	0.27	0.0079 (0.63)
PM_10_ (adjusted)	Weighted median	0.10	1.10	0.49–2.47	0.81		
	Inverse variance weighted	0.27	1.30	0.72–2.36	0.38	0.31	

OR, odds ratio; CI, confidence interval; IVW, inverse-variance weighted; PM, particulate matter; ASD, autism spectrum disorder

*P < 0.05

In the initial analysis, we did not observe a causal association between PM_2.5_ and ASD using the IVW method (β = 1.51, OR = 4.54, 95% CI: 0.55–37.67, *P* = 0.16) (Figure S1). However, significant heterogeneity was detected in sensitivity analyses (Table S2). To address the issue of significant heterogeneity, we identified an outlier SNP rs6749467 through the MR-PRESSO method, and subsequently excluded this SNP. After this adjustment, we found that genetically predicted PM_2.5_ increased the risk of ASD (IVW: β = 2.41, OR = 11.13, 95% CI: 2.54–48.76, *P* < 0.01; WM: β = 2.60, OR = 13.47, 95% CI: 3.14–57.82, *P* < 0.01). However, the MR-Egger method did not reveal a significant association between PM_2.5_ and ASD risk (*P* = 0.22) (Figure S2). This might be due to the potential horizontal pleiotropy allowed by this method. In the sensitivity analyses, Cochran’s Q test indicated the absence of significant heterogeneity in our adjusted analysis (*P* = 0.08) (Table S2). The MR-Egger’s intercept did not suggest a significant horizontal pleiotropy (intercept = -0.0295, *P* = 0.53), and no additional outliers were detected using MR-PRESSO method after adjustment for heterogeneity (global *P* = 0.16) (Table S3). Both leave-one-out analysis and funnel plot corroborated the stability of our results (Figure S2). The power value for PM_2.5_ and ASD is 1.00, which suggested that our MR analysis was robust (Table S4).

### Mendelian randomization analysis for causal association of PM_2.5_ absorbance and ASD

In the MR analysis assessing the relationship between PM_2.5_ absorbance and ASD risk, a significant increase in ASD risk was observed in the IVW method (β = 1.54, OR = 4.67, 95% CI: 1.21–18.01, *P* = 0.03) (Figure S3). However, no statistically significant associations were observed with the WM method and the MR-Egger method. Considering that over 50% of the IVs in our study were valid and the absence of significant horizontal pleiotropy, we deemed the IVW result was robust. In the sensitivity analyses, there was no evidence of significant heterogeneity in the association between PM_2.5_ absorbance and ASD risk (Cochran’s Q test *P* = 0.20), as shown in Table S2. The MR-Egger’s intercept did not indicate significant horizontal pleiotropy (intercept = 0.0123, *P* = 0.76). No outliers or pleiotropy were identified using the MR-PRESSO method (global *P* = 0.27). The leave-one-out analysis affirmed that the direction of our results was not influenced by any specific SNP. A symmetrical funnel plot further supported the absence of heterogeneity in our findings. The power value for PM_2.5_ absorbance and ASD is 1.00, which confirmed the robustness of our MR analyses (Table S4).

### Mendelian randomization analysis for causal association of PM_10_ and ASD

We found no causal association between PM_10_ and ASD risk (β = 0.47, OR = 1.60, 95% CI: 0.79–3.24, *P* = 0.19) (Figure S4). However, a significant heterogeneity was identified according to Cochran’s Q test (*P* = 0.04) (Table S2). After excluding the outlier SNP rs2248162, the IVW method (β = 0.27, OR = 1.30, 95% CI: 0.72–2.36, *P* = 0.38), the WM method (β = 0.10, OR = 1.10, 95% CI: 0.49–2.47, *P* = 0.81), and the MR-Egger method (β = -0.16, OR = 0.85, 95% CI: 0.14–5.14, *P* = 0.86) still showed no significant causal relationship between PM_10_ and ASD risk. We did not find significant heterogeneity and horizontal pleiotropy in the sensitivity analyses After the removal of outliers (Table S2, S3). Furthermore, the leave-one-out analysis and funnel plot suggested the robustness of our results (Figure S5). The power value for PM_10_ and ASD was 0.95, which indicated the reliable of our MR results (Table S4).

### Results from FDR-corrected analyses

Since multiple analyses of the same GWAS dataset, we employed the FDR correction. The results of FDR corrected q-values were 0.0022 for PM_2.5_, 0.0283 for PM_2.5_ absorbance, and 0.3065 for PM_10_. The causal associations between PM_2.5_ and PM_2.5_ absorbance with ASD remain significant after FDR correction.

## Discussion

In this study, we used genetic variants as IVs to investigate the causal relationship between PM and the risk of ASD. Our results indicated that PM_2.5_ and PM_2.5_ absorbance might increase the risk of ASD. However, no causal association was observed between PM_10_ and ASD risk.

The reduction of air pollution has profound implications for public health, significantly enhancing respiratory, cardiovascular and neurological well-being across the general population, reducing healthcare costs, and contributing to overall societal health resilience [6]. Within this broader context of improved general health, the specific impact on neurodevelopmental disorders, particularly ASD, is notable. Recent comprehensive meta-analyses analyzed 28 studies, aggregating data from over 750,000 newborns, revealing that every increase of 5 µg/m^3^ in PM_2.5_ consistently corresponded with a heightened risk of ASD across all analytical models. This risk was notably higher in relation to PM_2.5_ exposure when compared to other pollutants like PM_10_, NOx, or solvents [14]. Several population-based investigations, like the study in Southern California involving 294,937 mother-child pairs, indicated that increased PM_2.5_ exposure during the initial two trimesters was associated with a higher risk of ASD. The study went on to further emphasize stronger associations in male offspring compared to female [39]. Additional research echoes these findings, pinpointing both prenatal and postnatal PM_2.5_ exposures as risk factors for ASD in various geographical locations, ranging from Southwestern Pennsylvania to Israel [40–42]. The meticulous examination of large datasets further confirmed this association. In Southern California, analyzing 318,750 mother-child pairs from 2001 to 2014, prenatal exposure to key components of PM_2.5_ was linked to an increased risk of ASD in offspring [43]. Similarly, a multi-site case-control study conducted on United States children born between 2003 and 2006 revealed an association between early life PM_2.5_ exposure and ASD, further quantifying the risk with an odds ratio of 1.3 per 1.6 µg/m^3^ increase [44]. Yet, it is interesting to note that a cohort study in South Korea provided evidence suggesting both PM_2.5_ and PM_10_ exposures during the 4–10 trimester phase of pregnancy correlated with the onset of ASD [45]. However, not all studies have found a direct association between PM and ASD. For instance, a collaborative study across various European countries, which observed 8,079 children, found no significant relationship between prenatal exposure to pollutants, including PM and NO_2_, and childhood autistic traits [12]. Another study of 132,256 births highlighted an association with prenatal NO_x_ exposure but didn’t establish a significant link for PM_2.5_ [13]. As to animal experiments, neonatal Sprague-Dawley rats exhibited ASD-like behavioral characteristics upon early neonatal exposure to PM_2.5_ [46]. Additionally, another experiment showed that gestational and early-life exposure to PM_2.5_ led to notable behavioral and cognitive shifts in the offspring of juvenile male rats, underscoring the possible etiological role of PM_2.5_ in the onset of ASD and related conditions [47]. In light of these diverse findings, our research aligns with the predominant narrative suggesting the genetic association between PM_2.5_ and elevated ASD, and provided the evidence that PM_10_ was not significantly related to ASD.

The underlying mechanism connecting PM_2.5_ exposure and neurodevelopmental outcomes, including the potential risk for ASD, is complex and multi-faceted. The following are possible potential mechanisms: PM_2.5_ contains a mixture of fine particles and droplets that consist of acids, organic chemicals, metals, and soil or dust. Once inhaled, these particles can lead to the release of pro-inflammatory cytokines and reactive oxygen species [48, 49]. Chronic inflammation and oxidative stress can have harmful effects on both the mother and the fetus during pregnancy [47, 50, 51]. It’s believed that excessive inflammation, especially during critical periods of fetal brain development, can lead to altered neural connectivity and increased susceptibility to ASD. In animal models, maternal immune activation has been shown to lead to behavioral and brain abnormalities in offspring, which are reminiscent of human neurodevelopmental disorders [52]. Moreover, PM_2.5_ can penetrate the blood-brain barrier (BBB), either directly through the olfactory bulb or through systemic circulation. Once in the brain, these particles can cause local inflammation [53]. Neuroinflammation can disrupt the normal function and development of neural circuits, leading to abnormal patterns of neural connectivity and functionality associated with ASD [54]. In the presence of chronic neuroinflammation, microglial cells can become overactive and produce inflammatory mediators that affect brain development [55]. Furthermore, some components in PM_2.5_, particularly polycyclic aromatic hydrocarbons (PAHs), are known to disrupt endocrine function [56]. Prenatal exposure to certain endocrine disruptors has been shown to result in changes in social behavior, a core feature of ASD [57]. Finally, epigenetic mechanisms control gene expression without altering the underlying DNA sequence. PM_2.5_ exposure can lead to changes in DNA methylation patterns, histone modifications, and non-coding RNAs [58–60]. Altered epigenetic regulation can influence brain development and function, potentially leading to an increased risk of neurodevelopmental disorders [61, 62]. Recent studies have highlighted potential epigenetic modifications associated with ASD, suggesting this as a possible mechanism linking environmental exposures like PM_2.5_ to the disorder [62].

Urbanization and industrialization, while driving economic growth, pose significant challenges to global air quality [63]. To address these challenges, scientific research plays a pivotal role in unraveling the complex interplay between air pollution and health, offering vital insights that inform strategies to combat the adverse effects of poor air quality. Current scientific efforts are directed towards both mitigation and adaptation. On the mitigation front, advancements in emission reduction technologies are critical. This includes the development of cleaner fuel sources, such as renewable energy, and the promotion of energy-efficient practices in industries and households [64, 65]. Urban planning can also play a role by encouraging the use of public transportation, developing green spaces, and implementing zoning regulations that limit industrial activity in residential areas [66]. For adaptation, enhancing air quality monitoring systems is vital to provide real-time data and enable prompt responses to pollution incidents. Public health initiatives that increase awareness about the impact of air pollution and promote behavioral changes, such as reducing car usage or advocating for cleaner cooking and heating solutions, are also essential [67]. These strategies and methods could serve as references for various countries to adapt and apply according to their specific circumstances.

Our study has several strengths. The primary advantage is the employment of the MR design, it can mitigate confounding factors and reverse causation, and mimic randomized controlled trials. Secondly, we used the latest and largest GWAS database and rigorous screening to ensure the validity of the IVs, no significant heterogeneity and horizontal pleiotropy was found in the sensitivity analyses, and the Power value greater than 0.8 was also suggested the reliable of our study. Thirdly, the datasets utilized were of European populations, thereby minimizing the potential bias attributed to population stratification. Our research also presents several limitations. We did not assess the impact of PM_2.5_ on specific ASD subtypes, primarily attributable to the lack of the GWAS database for these subtypes. Additionally, despite leveraging the largest GWAS database available, the inclusion of SNPs significantly associated with PM_2.5_ was still limited. Lastly, we only obtained summary-level GWAS data and therefore could not analyze the detailed demographic information.

## Conclusion

In conclusion, our findings present strong genetic evidence of the association between PM_2.5_ and increased risk of ASD. As urbanization and industrial activities continue to burgeon, it becomes imperative to address air pollution as a modifiable risk factor. Implementing effective strategies to mitigate pollutants can potentially reduce the incidence of ASD, providing a clearer, healthier future for subsequent generations.

### Electronic supplementary material

Below is the link to the electronic supplementary material.

**Supplementary Material 1:** Detailed information for the investigation into the effects of PM on ASD risk using MR analyses

## Data Availability

No datasets were generated or analysed during the current study.
