# Supplementary material for: Particulate matter 2.5 causally increased genetic risk of autism spectrum disorder
Source: BMC Psychiatry. 2024 Feb 16;24:129. doi: 10.1186/s12888-024-05564-y (PMC10870670; doi:10.1186/s12888-024-05564-y)
Supplement: Supplementary file 1 — Supplementary Material 1: Detailed information for the investigation into the effects of PM on ASD risk using MR analyses [file 12888_2024_5564_MOESM1_ESM.docx]

Supplementary Materials

**Supplementary Table 1.** Characteristics of instrumental variables for particulate matter air pollution.

**Supplementary Table 2.** Heterogeneity of MR analysis for particulate matter air pollution on autism spectrum disorder risk.

**Supplementary Table 3.** Associations of genetic prediction of particulate matter air pollution on autism spectrum disorder risk in the MR-PRESSO analysis.

**Supplementary Table 4.** Power calculations for particulate matter air pollution on autism spectrum disorder risk.

**Supplementary Figure 1.** The MR estimates and sensitivity analyses between PM_2.5_ and ASD without adjustment for MR-PRESSO.

**Supplementary Figure 2.** The MR estimates and sensitivity analyses between PM_2.5_ and ASD after adjustment for MR-PRESSO.

**Supplementary Figure 3.** The MR estimates and sensitivity analyses between PM_2.5_ absorbance and ASD.

**Supplementary Figure 4.** The MR estimates and sensitivity analyses between PM_10_ and ASD without adjustment for MR-PRESSO.

**Supplementary Figure 5.** The MR estimates and sensitivity analyses between PM_10_ and ASD after adjustment for MR-PRESSO.

**Supplementary Table 1.** Characteristics of instrumental variables for particulate matter air pollution.

|  | Trait | SNP | Samplesize | EA | OA | SE | β | EAF | P | R^2^* | F - statistic** |
| --- | --- | --- | --- | --- | --- | --- | --- | --- | --- | --- | --- |
| 1 | PM_2.5_ | rs6749467 | 423,796 | A | G | 0.0022 | -0.0124 | 0.4658 | 1.40E-08 | 7.64E-05 | 32.39 |
| 2 | PM_2.5_ | rs1372504 | 423,796 | A | G | 0.0022 | 0.0123 | 0.3743 | 3.10E-08 | 7.08E-05 | 29.99 |
| 3 | PM_2.5_ | rs12203592 | 423,796 | T | C | 0.0026 | 0.0217 | 0.2129 | 6.20E-17 | 1.57E-04 | 66.68 |
| 4 | PM_2.5_ | rs114708313 | 423,796 | T | A | 0.0045 | 0.0246 | 0.0659 | 4.20E-08 | 7.42E-05 | 31.45 |
| 5 | PM_2.5_ | rs77255816 | 423,796 | T | C | 0.0057 | 0.0314 | 0.0365 | 4.20E-08 | 6.93E-05 | 29.38 |
| 6 | PM_2.5_ | rs77205736 | 423,796 | T | C | 0.0024 | 0.0135 | 0.2739 | 2.10E-08 | 7.27E-05 | 30.82 |
| 7 | PM_2.5_ | rs1537371 | 423,796 | A | C | 0.0021 | 0.0124 | 0.5001 | 8.50E-09 | 7.65E-05 | 32.43 |
| 8 | PM_2.5_ | rs72642437 | 423,796 | T | C | 0.0191 | 0.1134 | 0.0039 | 3.10E-09 | 9.89E-05 | 41.93 |
|  |  |  |  |  |  |  |  |  |  |  |  |
| 1 | PM_2.5_ absorbance | rs4915350 | 423,796 | C | A | 0.0079 | 0.0462 | 0.0187 | 5.70E-09 | 7.82E-05 | 33.14 |
| 2 | PM_2.5_ absorbance | rs12203592 | 423,796 | T | C | 0.0026 | 0.0166 | 0.2129 | 1.20E-10 | 9.19E-05 | 38.95 |
| 3 | PM_2.5_ absorbance | rs79475047 | 423,796 | C | T | 0.0066 | 0.0397 | 0.0268 | 1.60E-09 | 8.25E-05 | 34.99 |
| 4 | PM_2.5_ absorbance | rs59727727 | 423,796 | C | T | 0.0032 | 0.0180 | 0.1219 | 2.80E-08 | 6.94E-05 | 29.42 |
| 5 | PM_2.5_ absorbance | rs77205736 | 423,796 | T | C | 0.0024 | 0.0131 | 0.2739 | 4.50E-08 | 6.81E-05 | 28.87 |
|  |  |  |  |  |  |  |  |  |  |  |  |
| 1 | PM_10_ | rs182549 | 455,314 | T | C | 0.0022 | -0.0124 | 0.7388 | 2.10E-08 | 5.96E-05 | 27.12 |
| 2 | PM_10_ | rs114789974 | 455,314 | A | C | 0.0096 | -0.0552 | 0.0105 | 1.00E-08 | 6.31E-05 | 28.75 |
| 3 | PM_10_ | rs56084453 | 455,314 | G | A | 0.0024 | 0.0149 | 0.2097 | 5.90E-10 | 7.39E-05 | 33.67 |
| 4 | PM_10_ | rs13084230 | 455,314 | T | C | 0.0025 | -0.0136 | 0.2003 | 3.50E-08 | 5.89E-05 | 26.83 |
| 5 | PM_10_ | rs6793835 | 455,314 | A | G | 0.0022 | -0.0130 | 0.2638 | 6.60E-09 | 6.55E-05 | 29.82 |
| 6 | PM_10_ | rs4833095 | 455,314 | C | T | 0.0024 | 0.0251 | 0.2069 | 1.70E-25 | 2.07E-04 | 94.25 |
| 7 | PM_10_ | rs13122455 | 455,314 | T | C | 0.0025 | -0.0140 | 0.2000 | 1.30E-08 | 6.28E-05 | 28.57 |
| 8 | PM_10_ | rs6867849 | 455,314 | T | A | 0.0052 | -0.0315 | 0.0405 | 1.80E-09 | 7.70E-05 | 35.04 |
| 9 | PM_10_ | rs142169179 | 455,314 | A | G | 0.0073 | 0.0402 | 0.0203 | 4.40E-08 | 6.42E-05 | 29.22 |
| 10 | PM_10_ | rs2248162 | 455,314 | C | T | 0.0020 | 0.0118 | 0.6399 | 7.80E-09 | 6.44E-05 | 29.31 |
| 11 | PM_10_ | rs140295641 | 455,314 | A | T | 0.0062 | -0.0351 | 0.0274 | 1.30E-08 | 6.57E-05 | 29.91 |
| 12 | PM_10_ | rs9640029 | 455,314 | T | C | 0.0020 | -0.0138 | 0.4782 | 2.70E-12 | 9.51E-05 | 43.29 |
| 13 | PM_10_ | rs61620752 | 455,314 | G | T | 0.0028 | 0.0161 | 0.1483 | 6.40E-09 | 6.52E-05 | 29.71 |
| 14 | PM_10_ | rs2004679 | 455,314 | C | T | 0.0021 | 0.0119 | 0.3077 | 2.50E-08 | 6.05E-05 | 27.54 |
| 15 | PM_10_ | rs61875074 | 455,314 | C | A | 0.0038 | 0.0223 | 0.0730 | 6.80E-09 | 6.71E-05 | 30.56 |
| 16 | PM_10_ | rs147895162 | 455,314 | C | T | 0.0081 | -0.0448 | 0.0151 | 3.40E-08 | 5.97E-05 | 27.20 |
| 17 | PM_10_ | rs10498638 | 455,314 | C | T | 0.0025 | 0.0140 | 0.1883 | 3.30E-08 | 6.00E-05 | 27.34 |
| 18 | PM_10_ | rs74805019 | 455,314 | C | G | 0.0055 | -0.0307 | 0.0337 | 2.10E-08 | 6.15E-05 | 27.98 |
| 19 | PM_10_ | rs74247887 | 455,314 | T | C | 0.0059 | 0.0371 | 0.0287 | 2.70E-10 | 7.69E-05 | 35.03 |
| 20 | PM_10_ | rs4788565 | 455,314 | A | G | 0.0040 | -0.0219 | 0.0668 | 4.10E-08 | 5.99E-05 | 27.27 |
| 21 | PM_10_ | rs7200852 | 455,314 | A | C | 0.0045 | -0.0244 | 0.0547 | 4.00E-08 | 6.17E-05 | 28.12 |
| 22 | PM_10_ | rs60304336 | 455,314 | T | G | 0.0050 | 0.0279 | 0.0409 | 2.80E-08 | 6.12E-05 | 27.87 |

SNP: single nucleotide polymorphisms; EA: effect allele; OA: other allele; EAF: effect allele frequency; SE, standard error.

NOTE: *R^2^ = [2×β^2^×EAF×(1−EAF)]/[2×β^2^×EAF×(1−EAF) + (SE×β)^2^×2×n×EAF×(1 −EAF)]; **F - statistic=[R^2^×(n-2)]/(1-R^2^). R^2^, the proportion of exposed variability explained by individual genetic instrument; EAF, the effect allele frequency; β, the estimated effect of SNP; n, the sample size of the exposure of GWAS.

**Supplementary Table 2.** Heterogeneity of MR analysis for particulate matter air pollution on autism spectrum disorder risk.

| **Exposures** | **Outcome** | **Method** | **Q** | **Q_P value** |
| --- | --- | --- | --- | --- |
| PM_2.5_ | ASD | MR-Egger | 19.50 | <0.01* |
|  |  | IVW | 25.34 | <0.01* |
| PM_2.5_ (adjusted) | ASD | MR-Egger | 7.04 | 0.07 |
|  |  | IVW | 8.21 | 0.08 |
| PM_2.5_ absorbance | ASD | MR-Egger | 5.80 | 0.12 |
|  |  | IVW | 6.02 | 0.20 |
| PM_10_ | ASD | MR-Egger | 26.39 | 0.05* |
|  |  | IVW | 28.87 | 0.04* |
| PM_10_ (adjusted) | ASD | MR-Egger | 17.92 | 0.27 |
|  |  | IVW | 18.22 | 0.31 |

IVW, inverse-variance weighted; PM, particulate matter; ASD, autism spectrum disorder.

**Supplementary Table 3.** Associations of genetic prediction of particulate matter air pollution on autism spectrum disorder risk in the MR-PRESSO analysis.

| **Exposures** | **Outcome** | **MR-PRESSO** | | | | |
| --- | --- | --- | --- | --- | --- | --- |
|  |  | **Causal Estimate** | **SD** | **T-stat** | **P value** | **Global P value** |
| PM_2.5_ | ASD | 1.51 | 1.08 | 1.40 | 0.22 | <0.01* |
| PM_2.5_ (adjusted) | ASD | 2.41 | 0.75 | 3.2 | 0.03 | 0.16 |
| PM_2.5_ absorbance | ASD | 1.54 | 0.69 | 2.24 | 0.09 | 0.27 |
| PM_10_ | ASD | 0.47 | 0.36 | 1.31 | 0.21 | 0.04* |
| PM_10_ (adjusted) | ASD | 0.27 | 0.30 | 0.88 | 0.39 | 0.32 |

MR-PRESSO, MR-Pleiotropy Residual Sum and Outlier; SNP, single nucleotide polymorphisms; PM, particulate matter; ASD, autism spectrum disorder.

**Supplementary Table 4.** Power calculations for particulate matter air pollution on autism spectrum disorder risk.

| **Exposures** | **Outcome** | **Sample size** | **R^2^** | ***K*** | **OR** | **Power** |
| --- | --- | --- | --- | --- | --- | --- |
| PM_2.5_ | ASD | 423,796 | 6.96E-04 | 0.40 | 11.13 | 1.00 |
| PM_2.5_ absorbance | ASD | 423,796 | 3.90E-04 | 0.40 | 4.67 | 1.00 |
| PM_10_ | ASD | 455,314 | 1.59E-03 | 0.40 | 1.30 | 0.95 |

OR, odds ratio; PM, particulate matter; ASD, autism spectrum disorder. *K* refers to Proportion of cases in the study.


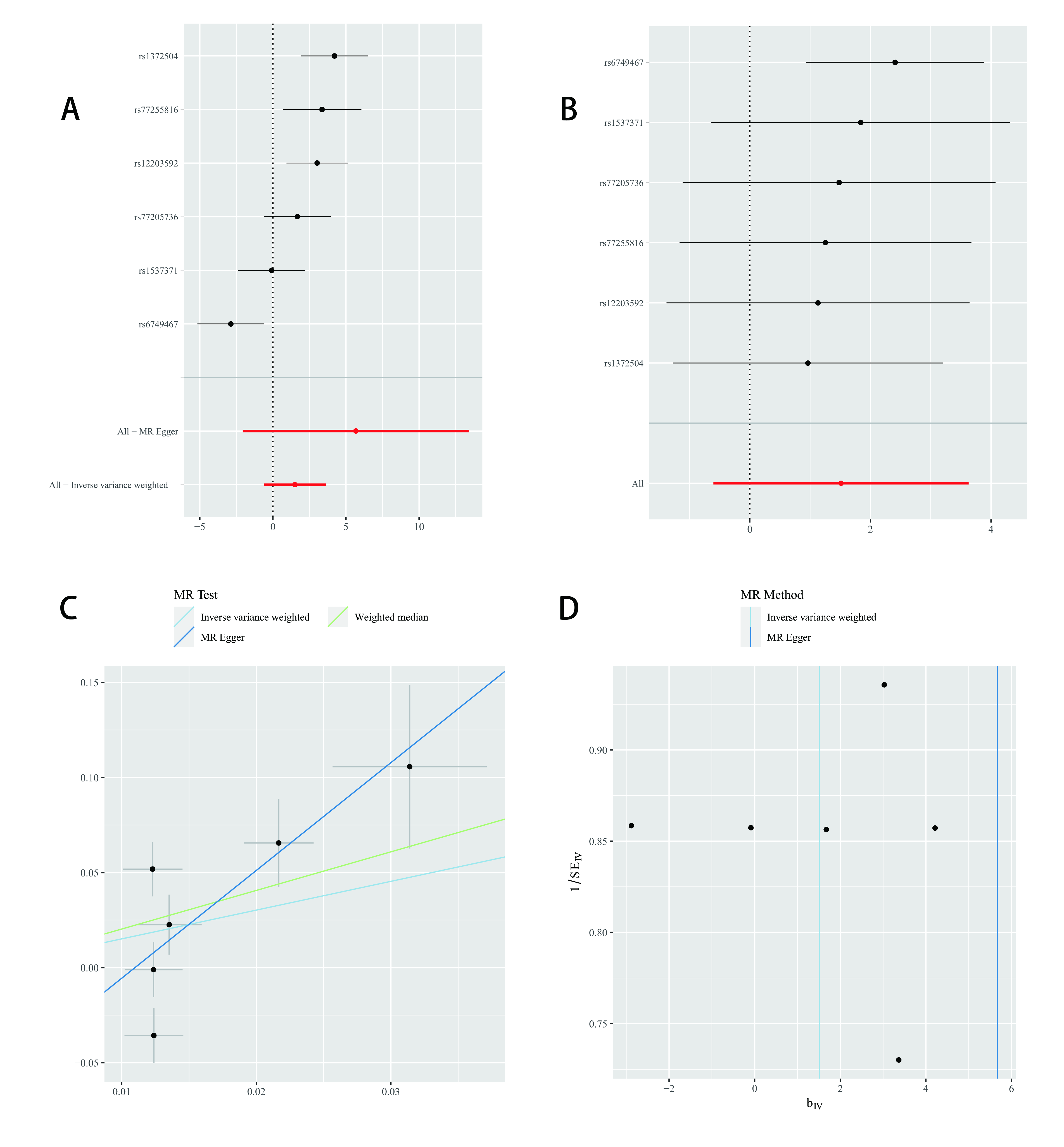


**Supplementary Figure 1.** The MR estimates and sensitivity analyses between PM_2.5_ and ASD without adjustment for MR-PRESSO. A) MR estimates illustrate the relationship between PM_2.5_ and the risk of ASD. This demonstrates no significant association between PM_2.5_ and ASD (OR = 4.54, 95% CI: 0.55-37.67, P = 0.16). B) The leave-one-out plot for PM_2.5_ and ASD highlights how causal estimates (represented as points with horizontal lines) change when individual SNPs are excluded. C) The scatter plot visualizing the association between PM_2.5_ and ASD displays three lines, each representing different MR methods: inverse-variance weighted (IVW), MR-Egger, and weighted median (WM). The MR-Egger's intercept stands at -0.0623 (P = 0.34), indicating no significant horizontal pleiotropy in our research. D) The funnel plot concerning PM_2.5_ and ASD is symmetrical, signifying the absence of polymorphism.


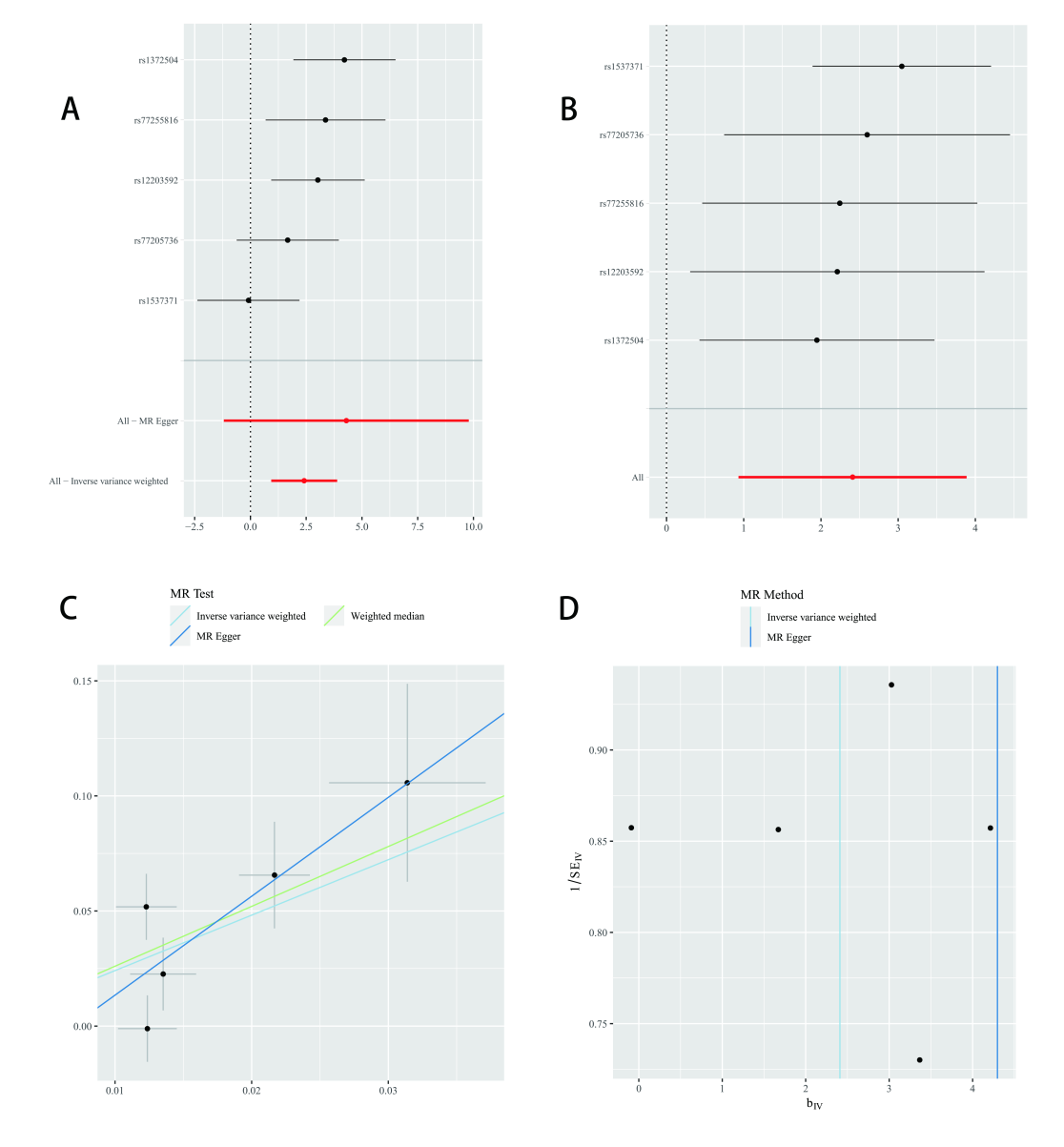


**Supplementary Figure 2.** The MR estimates and sensitivity analyses between PM_2.5_ and ASD after adjustment for MR-PRESSO. A) MR estimates illustrate the relationship between PM_2.5_ (adjusted) and ASD risk, indicating a significant association (OR = 11.13, 95%CI: 2.54-48.76, P < 0.01). B) The leave-one-out test for PM_2.5_ (adjusted) and ASD suggests that our results are stable. C) Scatter plot of the association between PM_2.5_ (adjusted) and ASD. The MR-Egger's intercept is -0.0295 (P = 0.53), suggesting that there is no significant horizontal pleiotropy in our study. Notably, the slopes across all three methods are directionally consistent, affirming the stability of our findings. D) The funnel plot on PM_2.5_ (adjusted) and ASD. The funnel plots are symmetric, which shows that the absence of polymorphism.


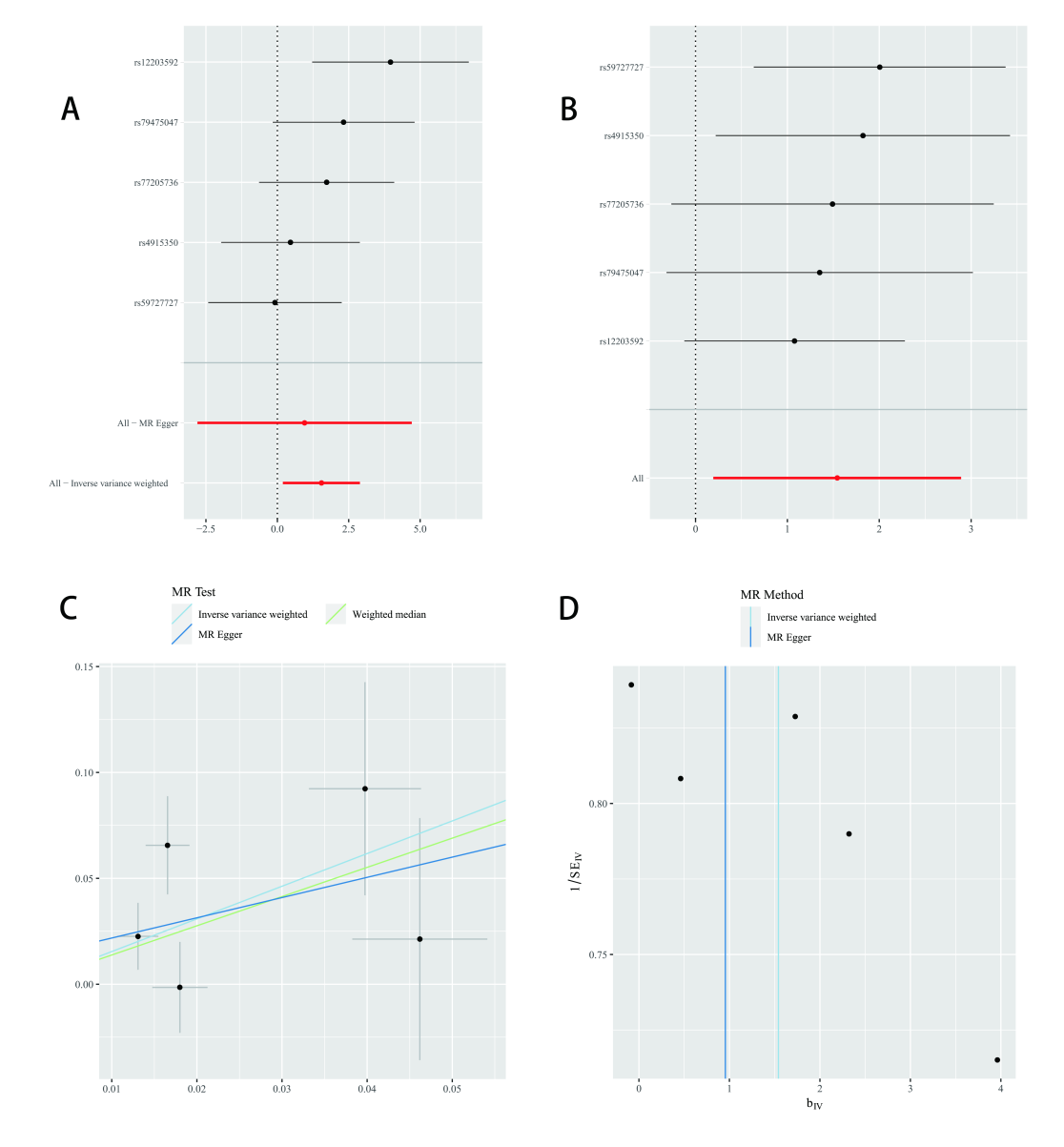


**Supplementary Figure 3.** The MR estimates and sensitivity analyses between PM_2.5_ absorbance and ASD. A) MR estimates of the association between PM_2.5_ absorbance and ASD. The figure shows that there is a significant association between PM_2.5_ absorbance and ASD (OR = 4.67, 95% CI: 1.21-18.01, P = 0.03). B) The leave-one-out plot of PM_2.5_ absorbance and ASD. C) The scatter plot depicts the association between PM_2.5_ absorbance and ASD. The intercept of MR-Egger registers at 0.0123 (P = 0.76), indicating an absence of significant horizontal pleiotropy in our research. D) The funnel plot on PM_2.5_ absorbance and ASD. The funnel plots are also symmetric, which shows that the absence of polymorphism.


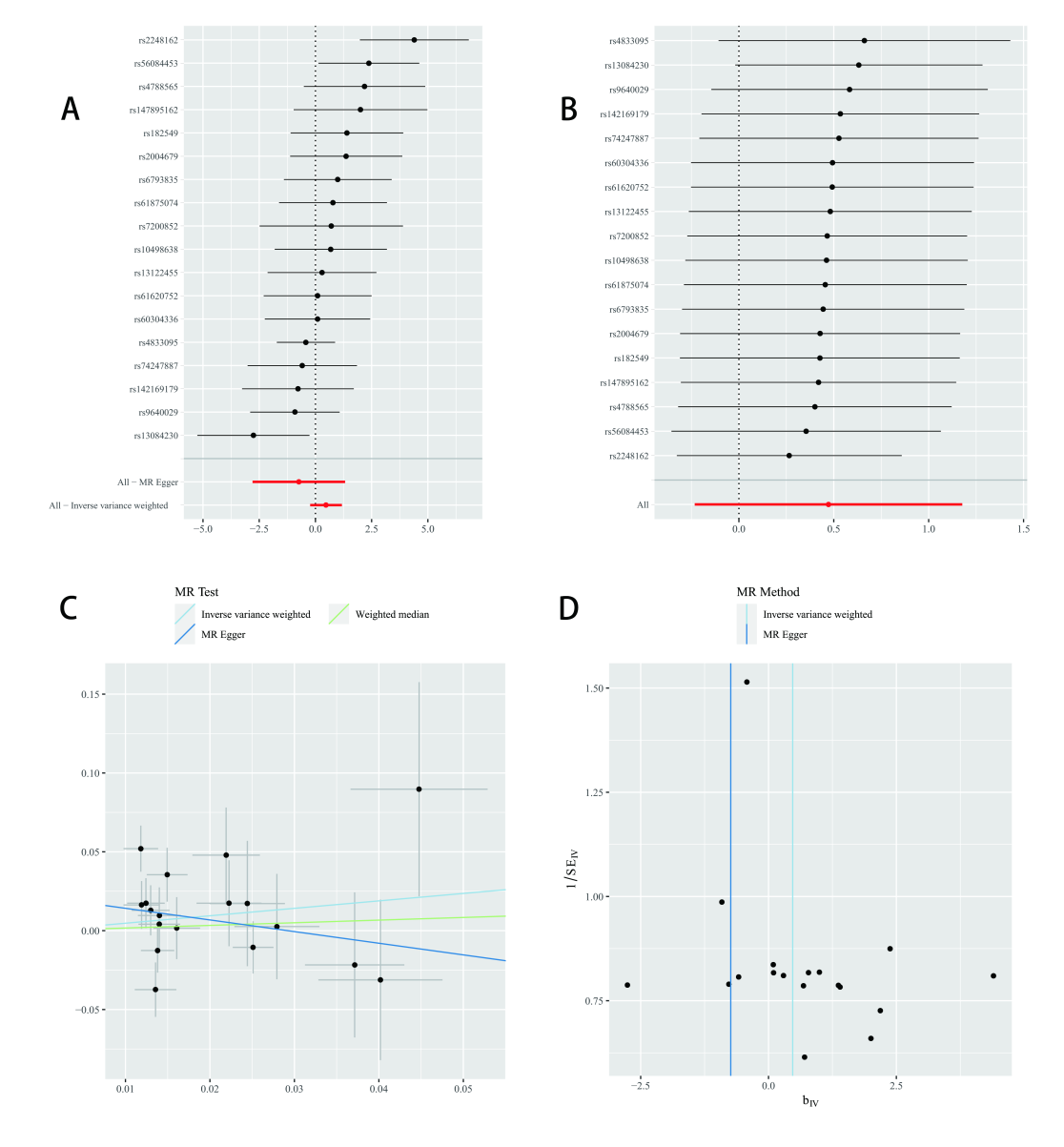


**Supplementary Figure 4.** The MR estimates and sensitivity analyses between PM_10_ and ASD without adjustment for MR-PRESSO. A) MR estimates demonstrate the relationship between PM_10_ and ASD, indicating no significant association (OR = 1.60, 95% CI: 0.79-3.24, P = 0.19). B) The leave-one-out plot concerning PM_10_ and ASD. This analysis confirms the consistency of our results. C) The scatter plot detailing the association between PM_10_ and ASD shows three distinct lines. The intercept of MR-Egger stands at 0.0215 (P = 0.24), indicating no substantial horizontal pleiotropy within our research. D) The funnel plot relating to PM_10_ and ASD is symmetrical, signifying the absence of polymorphism.


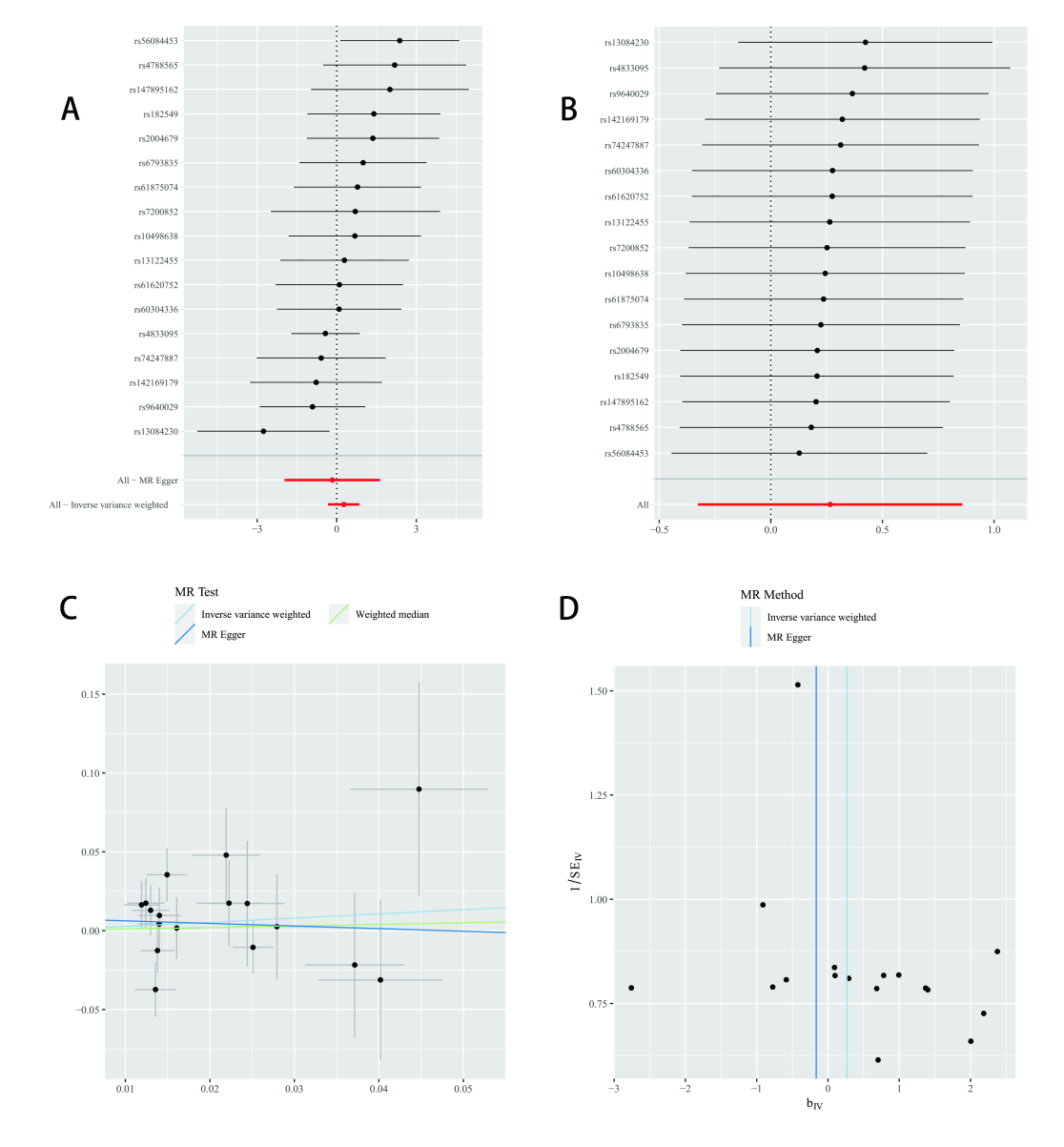


**Supplementary Figure 5.** The MR estimates and sensitivity analyses between PM_10_ and ASD after adjustment for MR-PRESSO. A) MR estimates depict the relationship between PM_10_ (adjusted) and ASD, indicating no significant association (OR = 1.30, 95% CI: 0.72-2.36, P = 0.38). B) The leave-one-out plot concerning PM_10_ (adjusted) and ASD confirms the consistency of our results. C) The scatter plot detailing the association between PM_10_ (adjusted) and ASD shows three distinct lines, representing different effect sizes. The intercept of MR-Egger stands at 0.0079 (P = 0.63), signifying an absence of notable horizontal pleiotropy in our research. D) The funnel plot for PM_10_ (adjusted) and ASD is symmetrical, denoting the absence of polymorphism.
